# Supplementary material for: Metformin treatment results in distinctive skeletal muscle mitochondrial remodeling in rats with different intrinsic aerobic capacities
Source: Aging Cell. 2024 Jun 24;23(9):e14235. doi: 10.1111/acel.14235 (PMC11488331; doi:10.1111/acel.14235)
Supplement: Supplementary file 1 — Figure S1. [file ACEL-23-e14235-s007.pdf]

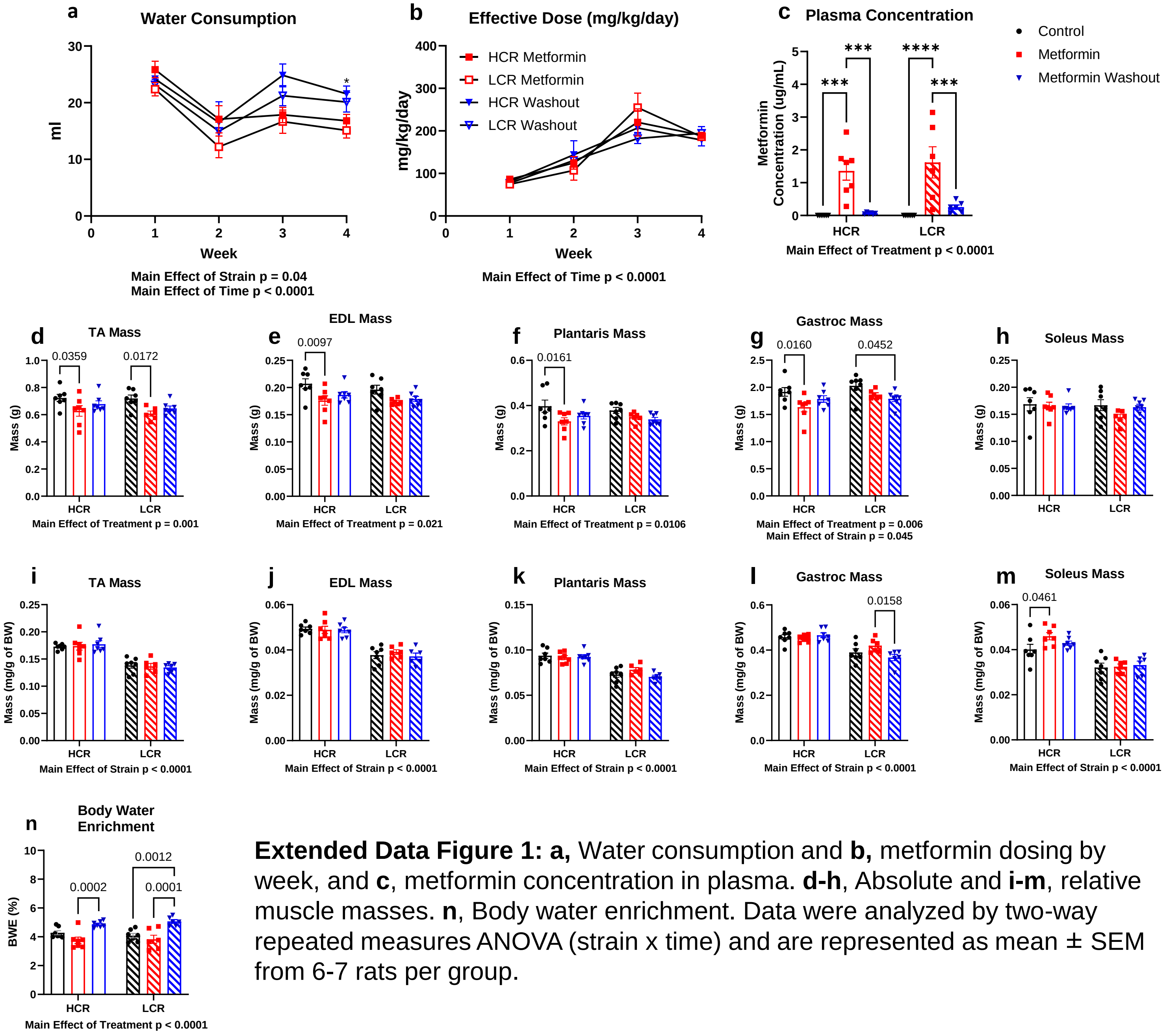

Extended Data Figure 1: a, Water consumption and **b,** metformin dosing by week, and **c**, metformin concentration in plasma. **d-h**, Absolute and **i-m**, relative muscle masses. **n**, Body water enrichment. Data were analyzed by two-way repeated measures ANOVA (strain x time) and are represented as mean ± SEM from 6-7 rats per group.
