# Supplementary material for: Metformin treatment results in distinctive skeletal muscle mitochondrial remodeling in rats with different intrinsic aerobic capacities
Source: Aging Cell. 2024 Jun 24;23(9):e14235. doi: 10.1111/acel.14235 (PMC11488331; doi:10.1111/acel.14235)
Supplement: Supplementary file 2 — Figure S2. [file ACEL-23-e14235-s001.pdf]

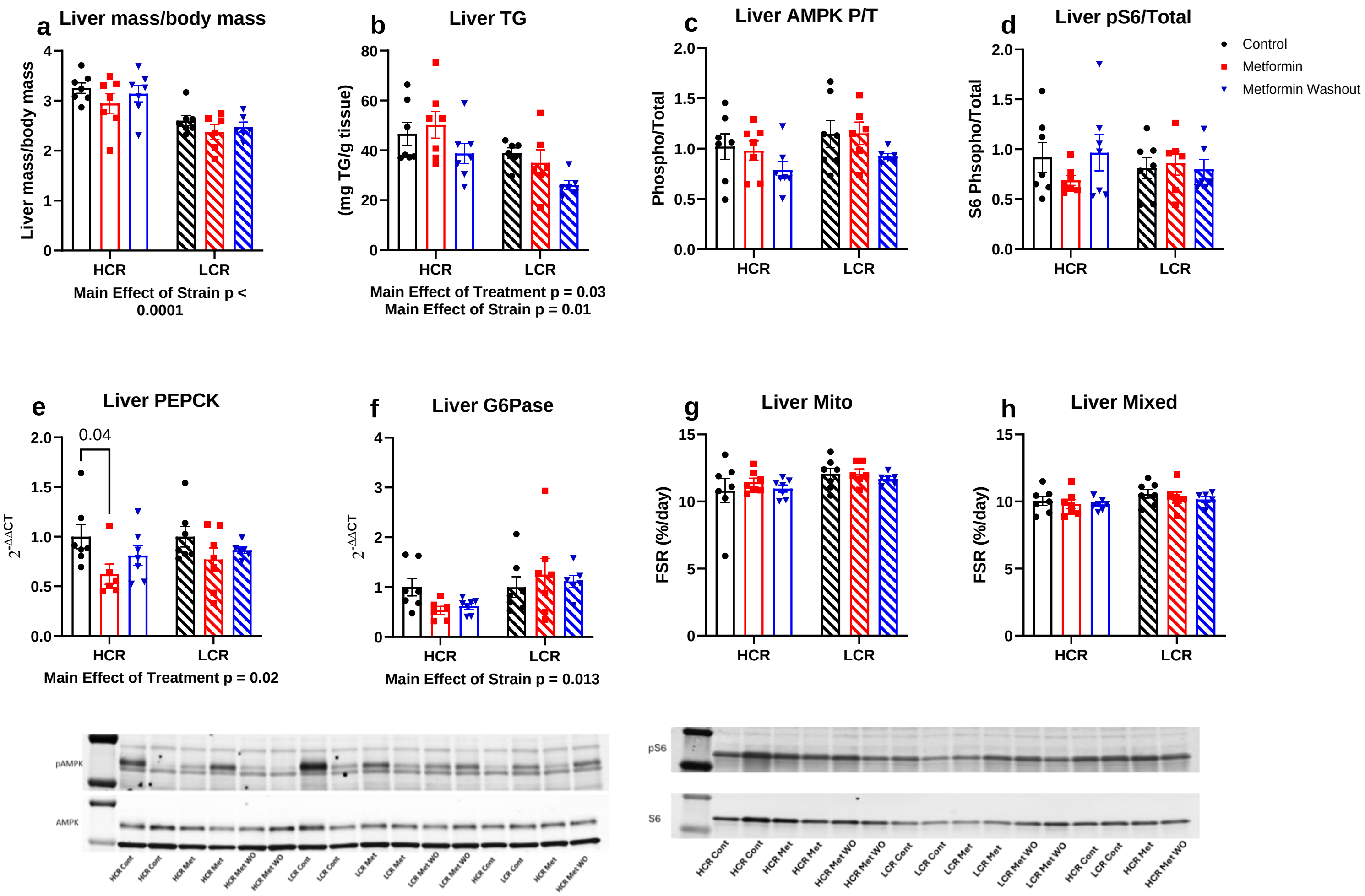

Extended Data Figure 2: a, Liver mass, **b**, triglyceride content, **c**, AMPK phospho (Thr172) to total ratio and **d**, S6 phospho (Ser235/236) to total ratio. Gene expression of liver **e**, PEPCK and **f**, G6Pase. Determination of the fractional synthesis rates in the **g**, mitochondrial and **h,** mixed fractions of liver. Data were analyzed by two-way ANOVA (strain x treatment) and are represented as mean ± SEM from 6-7 rats per group.
