# Supplementary material for: Metformin treatment results in distinctive skeletal muscle mitochondrial remodeling in rats with different intrinsic aerobic capacities
Source: Aging Cell. 2024 Jun 24;23(9):e14235. doi: 10.1111/acel.14235 (PMC11488331; doi:10.1111/acel.14235)
Supplement: Supplementary file 3 — Figure S3. [file ACEL-23-e14235-s003.pdf]

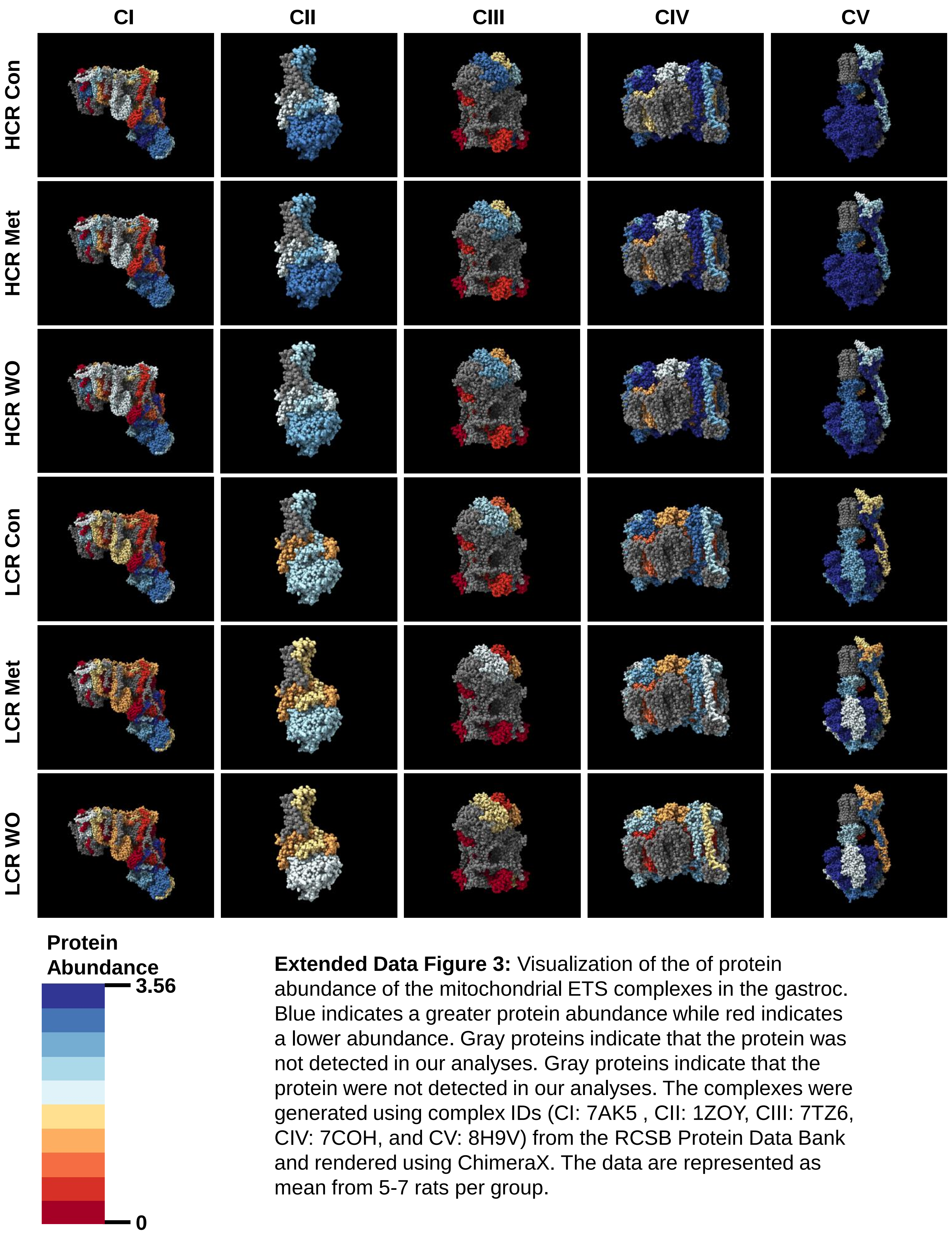

Extended Data Figure 3: Visualization of the of protein abundance of the mitochondrial ETS complexes in the gastroc. Blue indicates a greater protein abundance while red indicates a lower abundance. Gray proteins indicate that the protein was not detected in our analyses. Gray proteins indicate that the protein were not detected in our analyses. The complexes were generated using complex IDs (CI: 7AK5 , CII: 1ZOY, CIII: 7TZ6, CIV: 7COH, and CV: 8H9V) from the RCSB Protein Data Bank and rendered using ChimeraX. The data are represented as mean from 5-7 rats per group.
