# Supplementary material for: Metformin treatment results in distinctive skeletal muscle mitochondrial remodeling in rats with different intrinsic aerobic capacities
Source: Aging Cell. 2024 Jun 24;23(9):e14235. doi: 10.1111/acel.14235 (PMC11488331; doi:10.1111/acel.14235)
Supplement: Supplementary file 4 — Figure S4. [file ACEL-23-e14235-s002.pdf]

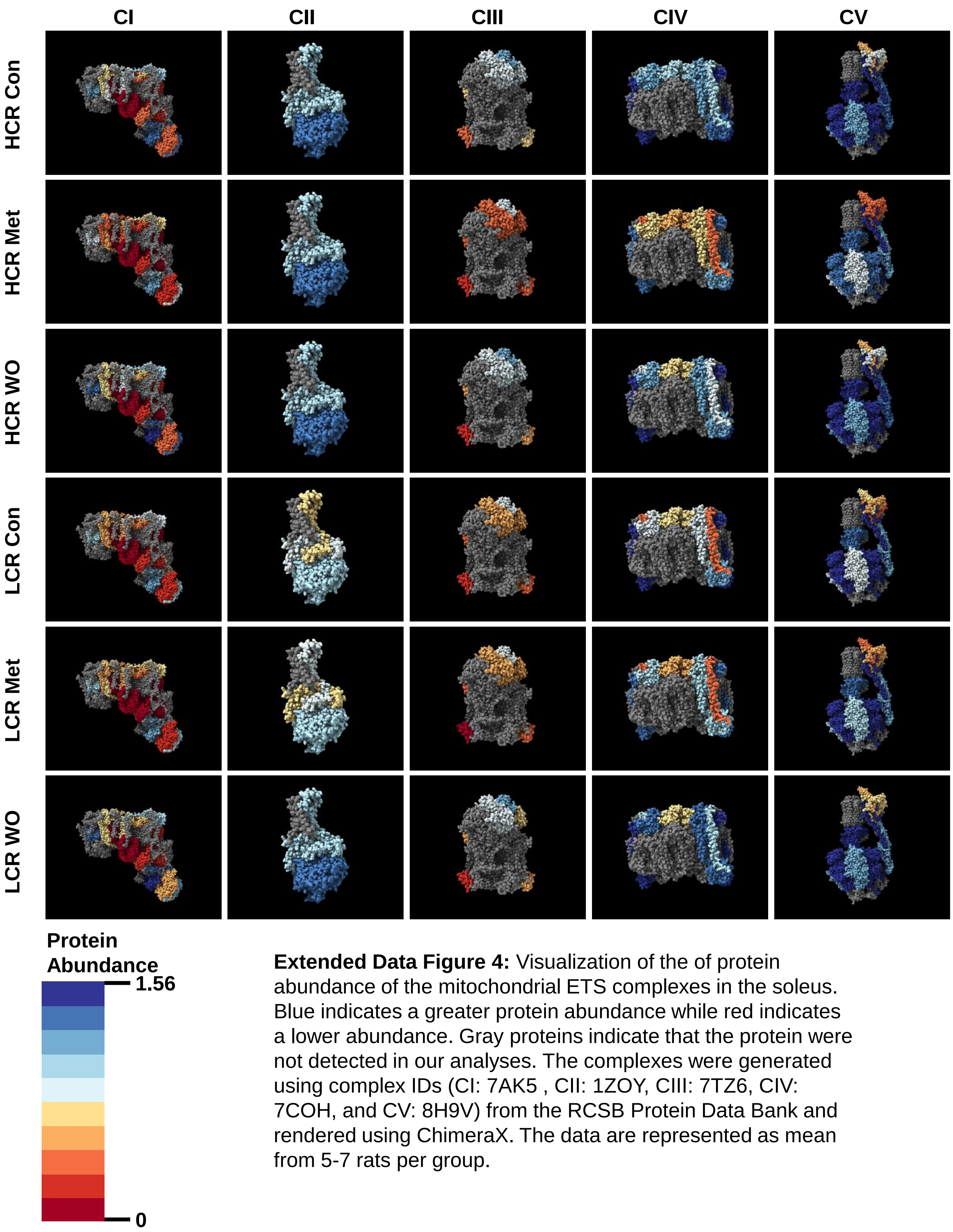

Extended Data Figure 4: Visualization of the of protein abundance of the mitochondrial ETS complexes in the soleus. Blue indicates a greater protein abundance while red indicates a lower abundance. Gray proteins indicate that the protein were not detected in our analyses. The complexes were generated using complex IDs (CI: 7AK5 , CII: 1ZOY, CIII: 7TZ6, CIV: 7COH, and CV: 8H9V) from the RCSB Protein Data Bank and rendered using ChimeraX. The data are represented as mean from 5-7 rats per group.
