# Supplementary material for: Metformin treatment results in distinctive skeletal muscle mitochondrial remodeling in rats with different intrinsic aerobic capacities
Source: Aging Cell. 2024 Jun 24;23(9):e14235. doi: 10.1111/acel.14235 (PMC11488331; doi:10.1111/acel.14235)
Supplement: Supplementary file 5 — Figure S5. [file ACEL-23-e14235-s004.pdf]

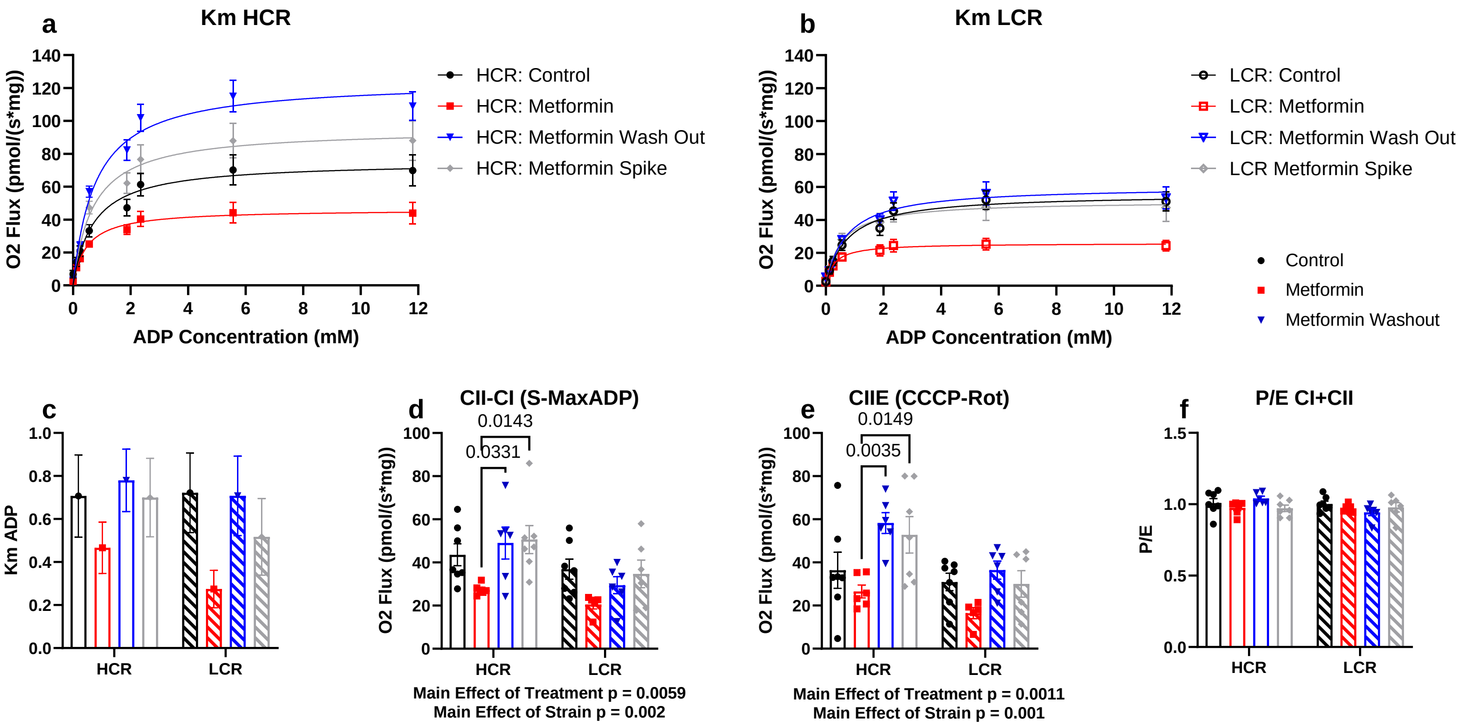

Extended Data Figure 5: ADP titration curve with metformin administration in **a**, HCR and **b**, LCRs. **c**, The apparent Km of ADP was calculated using Michaelis-Menten Kinetics. Contribution of **d**, Complex CII and **e**, Uncoupled respiration. **f**, The ratio of maximal coupled OXPHOS to uncoupled ETS (P/E) for the complex I+II. Data were analyzed by two-way ANOVA (group x treatment) and are represented as mean ± SEM from 6-7 rats per group.
