# Supplementary material for: Metformin treatment results in distinctive skeletal muscle mitochondrial remodeling in rats with different intrinsic aerobic capacities
Source: Aging Cell. 2024 Jun 24;23(9):e14235. doi: 10.1111/acel.14235 (PMC11488331; doi:10.1111/acel.14235)
Supplement: Supplementary file 6 — Table S1. [file ACEL-23-e14235-s005.pdf]

Complex 1		
Protein	Peptide	NEH
Ndufa2	AHPDLPILIR	20
Ndufa2	NVSLNNLSAAEVTK	25
Ndufa3	ATPYNYPVPVR	19
Ndufa4	LGPNEQYK	16
Ndufa4	FYSVNVDYSK	11
Ndufa5	KLENLLQGGEVEEVILQAEK	43
Ndufa5	LTILYTK	3
Ndufa6	VVDLLVIK	6
Ndufa6	FFHETETPRPK	20
Ndufa7	TAESSAVAATK	26
Ndufa7	EVVPPSIIMSSQK	25
Ndufa7	NWASGQDLQAK	25
Ndufa8	VSSAVLK	11
Ndufa8	LVNGCALNFFR	17
Ndufa9	NFDFEDVFVNIPR	20
Ndufa9	FLNHFANYR	15
Ndufa10	VVEDIEYLNYNK	17
Ndufa10	YGLLASILGDK	16
Ndufa11	LEGWELFATPK	18
Ndufa12	FNVSATPEQYVPYSTTR	30
Ndufb5	TLAILQIESEK	22
Ndufb6	FWNNFLR	8
Ndufb7	DYCAHYLIR	16
Ndufb10	ELEQFTQVSK	20
Ndufb10	VDQEIINIIQER	27
Ndufb10	RTPAPSPQTSIPNPITYLTK	35
Ndufb11	AVIAPSGVER	24
Ndufc2	TYAEILEPFHPVR	26
Nd1	YSLFGALR	14
Nd4	VSIILDPLTK	11
Nd5	STSITQTQLSK	18
Ndufs1	FEAPLFNAR	21
Ndufs1	VALIGSPVDLTYR	20
Ndufs2	VLFGEITR	12
Ndufs3	SLADLTAVDVPTR	23
Ndufs3	VVAEPVELAQEFR	32
Ndufs5	LGISLDR	12
Ndufs5	HFIFLSAEQPYK	23
Ndufs6	TGTCGYCGLQFK	15
Ndufs7	LDDLINWAR	15
Ndufs7	AHQSVAATGSPSSTQSAVSK	49
Ndufs7	QADVMIVAGTLTNK	22
Ndufv1	YLVVNADEGEPGTCK	26
Ndufv1	NACDSDYDFDVFVVR	23
Ndufv2	DSDSILETLQR	22
Ndufv2	AAAVLPVLDLAQR	30

Complex 2		
Protein	Peptide	NEH
SdhA	GEGGILINSQGER	30
SdhA	VSQLYGDLQHLK	20
SdhB	SIEPYLK	11
SdhB	DEFTEER	17
SdhC	NTSSNRPVSPHLTIYR	29

Complex 3		
Protein	Peptide	NEH
Cytb	DVNYGWLIR	11
Cyc1	GLLSSLDHTSIR	21
Uqcrc1	NNGAGYFLEHLAFK	25
Uqcrc1	SGMFWLR	10
Uqcrc1	NALISHLDGTTPVCEDIGR	35
Uqcrc2	ITSEELHYFVQNHFTSAR	35
Uqcrc2	NALANPLYCPDYR	25
Uqcrb	RLPENLYNDR	20
Uqcrh	SQTEEDCTEELFDFLHAR	40
Uqcrfs1	EIDQEAAVEVSQLR	38
Uqcrfs1	SGPFAPVLSATSR	28
Uqcr10	AFDQGADAVYDYINEGK	34
Uqcr10	LYSLLFR	8
Uqcr11	LILDWVPYINGK	13
Uqcc2	FAPTRPEEK	21
Ttc19	DEFSVQHIR	20

Complex 4		
Protein	Peptide	NEH
Mtco1	VFSWLATLHGGNIK	19
Mtco2	VVLPMELPIR	17
Mtco2	TDAIPGR	15
Cox4i1	DYPLPDVAHVK	18
Cox4i1	ADWSSLSR	17
Cox5a	LNDFASAVR	19
Cox5b	ASGGGVPTDEEQATGLER	44
Cox5b	GVGALAAQALR	29
Cox6a2	GDHGGAGANTWR	26
Cox6b1	TAPFDSR	15
Cox6b1	NCWQNYLDFHR	18
Cox6b1	GGDVSVCEWYR	19
Cox6b1	SLCPVSWVSAWDDR	25
Cox6b1	IAEGTFPGK	16
Cox6c	AYADFYR	14

Complex 5		
Protein	Peptide	NEH
Atp5a1	FESAFLSHVVSQHQSLLGNIR	43
Atp5a1	HALIIYDDLSK	17
Atp5a1	TSIAIDTIINQK	19
Atp5a1	TGTAEMSSILEER	29
Atp5b	FLSQPFQVAEVFTGHMGK	32
Atp5b	EGNDLYHEMIESGVINLK	32
Atp5c1	VYGTGSLALYEK	18
Atp5c1	LTLTFNR	7
Atp5c1	THSDQFLVSFK	16
Atp5d	AQSELSGAADEAAR	45
Atp5d	IEANEALVK	20
Atp5e	FSQICAK	14
Atp5f1	PLPPLPEYGGK	20
Atp5f1	NNIALALEVTYR	22
Atp5h	LASLSEKPPAIDWAYYR	35
Atp5h	SWNETFHTR	15
Atp5h	NCAQFVTGSQAR	28
Atp5i	VPPVQVSPLIK	18
Atp5i	ELAEAEDVSIFK	27
Atp5j2	DFTPSGIAGAFR	24
Atp5j2	ELPSWILMR	15
Atp5l	LATFWHYAR	15
Atp5o	LVRPPVQVYGIEGR	27

Supplemental Table 1: Validation peptides and number of exchangeable hydrogens (NEH).
