# Supplementary material for: Metformin treatment results in distinctive skeletal muscle mitochondrial remodeling in rats with different intrinsic aerobic capacities
Source: Aging Cell. 2024 Jun 24;23(9):e14235. doi: 10.1111/acel.14235 (PMC11488331; doi:10.1111/acel.14235)
Supplement: Supplementary file 7 — Table S2. [file ACEL-23-e14235-s008.pdf]

Gastroc Synthesis Rates (k(1/day))						
	HCR Con	HCR MET	HCR WO	LCR Con	LCR MET	LCR WO
**Ndufa2**	0.0308 ± 0.0100	0.0485 ± 0.0345	0.0313 ± 0.0074	0.0182 ± 0.0059	0.0387 ± 0.0208	0.0446 ± 0.0311
**Ndufa3**	0.0141 ± 0.0047	0.0183 ± 0.0061	0.0313 ± 0.0038 ‡	0.0199 ± 0.0072	0.0235 ± 0.0057	0.0198 ± 0.0046
**Ndufa4**	0.0428 ± 0.0133	0.0396 ± 0.0101	0.0505 ± 0.0102	0.0486 ± 0.0193	0.0554 ± 0.0154	0.0535 ± 0.0142
**Ndufa5**	0.0589 ± 0.0188	0.0523 ± 0.0399	0.0484 ± 0.0170	0.0465 ± 0.0245	0.0783 ± 0.0683	0.0586 ± 0.0276
**Ndufa6**	0.0614 ± 0.0160	0.0608 ± 0.0260	0.0868 ± 0.0420	0.0561 ± 0.0213	0.0574 ± 0.0285	0.1224 ± 0.0991 $
**Ndufa7**	0.0592 ± 0.0140	0.0828 ± 0.0744	0.0643 ± 0.0138	0.0747 ± 0.0368	0.0749 ± 0.0276	0.0827 ± 0.0244
**Ndufa8**	0.0276 ± 0.0061	0.0266 ± 0.0104	0.0268 ± 0.0049	0.0312 ± 0.0100	0.0239 ± 0.0089	0.0308 ± 0.0080
**Ndufa9**	0.0544 ± 0.0150	0.0567 ± 0.0083	0.0558 ± 0.0116	0.0537 ± 0.0118	0.0639 ± 0.0098	0.0548 ± 0.0147
**Ndufa10**	0.0595 ± 0.0108	0.0673 ± 0.0168	0.0618 ± 0.0138	0.0624 ± 0.0258	0.0601 ± 0.0112	0.0657 ± 0.0136
**Ndufa11**	0.0581 ± 0.0054	0.0703 ± 0.0090 $	0.0554 ± 0.0098 †	0.0607 ± 0.0071	0.0558 ± 0.0064	0.0775 ± 0.0127 ‡
**Ndufa12**	0.0440 ± 0.0050	0.0377 ± 0.0083	0.0406 ± 0.0041	0.0423 ± 0.0096	0.0376 ± 0.0116	0.0464 ± 0.0080
**Ndufb5**	0.0302 ± 0.0024	0.0146 ± 0.0043 $	0.0205 ± 0.0023 ‡	0.0222 ± 0.0033	0.0270 ± 0.0032 $	0.0195 ± 0.0046 †
**Ndufb6**	0.0172 ± 0.0128	0.0000 ± 0.0000 $	0.0120 ± 0.0047 †	0.0072 ± 0.0072	0.0073 ± 0.0062	0.0047 ± 0.0000
**Ndufb7**	0.0177 ± 0.0042	0.0118 ± 0.0001 $	0.0198 ± 0.0073 †	0.0133 ± 0.0001	0.0077 ± 0.0011 $	0.0162 ± 0.0047 †
**Ndufb10**	0.0256 ± 0.0090	0.0279 ± 0.0092	0.0299 ± 0.0062	0.0313 ± 0.0106	0.0215 ± 0.0138	0.0362 ± 0.0114 †
**Ndufb11**	0.0288 ± 0.0028	0.0200 ± 0.0036	0.0328 ± 0.0030 †	0.0282 ± 0.0044	0.0291 ± 0.0066	0.0280 ± 0.0033
**Ndufc2**	0.0263 ± 0.0043	0.0137 ± 0.0051 $	0.0261 ± 0.0018 †	0.0236 ± 0.0025	0.0195 ± 0.0025	0.0250 ± 0.0050 †
**Nd1**	0.0234 ± 0.0063	0.0000 ± 0.0000 $	0.0165 ± 0.0038 †	0.0182 ± 0.0061	0.0025 ± 0.0025 $	0.0162 ± 0.0082 †
**Nd4**	0.0298 ± 0.0048	0.0154 ± 0.0010 $	0.0259 ± 0.0058 †	0.0272 ± 0.0093	0.0162 ± 0.0001 $	0.0274 ± 0.0057 †
**Nd5**	0.0435 ± 0.0022	0.0427 ± 0.0028	0.0465 ± 0.0046	0.0491 ± 0.0046	0.0480 ± 0.0055	0.0542 ± 0.0053 †
**Ndufs1**	0.0591 ± 0.0055	0.0542 ± 0.0095	0.0606 ± 0.0062	0.0587 ± 0.0050	0.0627 ± 0.0090	0.0646 ± 0.0075
**Ndufs2**	0.0468 ± 0.0044	0.0482 ± 0.0078	0.0510 ± 0.0070	0.0410 ± 0.0044	0.0520 ± 0.0103 $	0.0553 ± 0.0040 $
**Ndufs3**	0.0490 ± 0.0067	0.0478 ± 0.0082	0.0540 ± 0.0077	0.0491 ± 0.0056	0.0566 ± 0.0078	0.0614 ± 0.0069 $
**Ndufs5**	0.0374 ± 0.0054	0.0343 ± 0.0117	0.0363 ± 0.0069	0.0344 ± 0.0108	0.0354 ± 0.0098	0.0331 ± 0.0098
**Ndufs6**	0.0921 ± 0.0172	0.0894 ± 0.0134	0.0886 ± 0.0067	0.0644 ± 0.0114	0.0740 ± 0.0185	0.1105 ± 0.0294 ‡
**Ndufs7**	0.0768 ± 0.0143	0.0713 ± 0.0264	0.0750 ± 0.0160	0.0667 ± 0.0190	0.0696 ± 0.0223	0.0712 ± 0.0210
**Ndufv1**	0.0499 ± 0.0092	0.0408 ± 0.0131	0.0483 ± 0.0119	0.0439 ± 0.0149	0.0604 ± 0.0444	0.0609 ± 0.0116
**Ndufv2**	0.0647 ± 0.0187	0.0677 ± 0.0279	0.0717 ± 0.0130	0.0733 ± 0.0208	0.0730 ± 0.0304	0.0718 ± 0.0125
**SdhA**	0.0548 ± 0.0086	0.0524 ± 0.0088	0.0540 ± 0.0045	0.0537 ± 0.0089	0.0650 ± 0.0163	0.0589 ± 0.0096
**SdhB**	0.0547 ± 0.0137	0.0629 ± 0.0181	0.0521 ± 0.0102	0.0721 ± 0.0213	0.0613 ± 0.0251	0.0631 ± 0.0239
**SdhC**	0.0410 ± 0.0028	0.0377 ± 0.0041	0.0441 ± 0.0075	0.0392 ± 0.0042	0.0295 ± 0.0028	0.0390 ± 0.0195
**Cytb**	0.0471 ± 0.0106	0.0311 ± 0.0058	0.0527 ± 0.0102 †	0.0371 ± 0.0115	0.0387 ± 0.0193	0.0563 ± 0.0228
**Cyc1**	0.0330 ± 0.0041	0.0334 ± 0.0040	0.0334 ± 0.0031	0.0399 ± 0.0055	0.0290 ± 0.0060 $	0.0499 ± 0.0071 ‡
**Uqcrc1**	0.0222 ± 0.0082	0.0265 ± 0.0082	0.0263 ± 0.0059	0.0303 ± 0.0137	0.0324 ± 0.0148	0.0359 ± 0.0160
**Uqcrc2**	0.0261 ± 0.0053	0.0216 ± 0.0096 $	0.0308 ± 0.0059 $	0.0357 ± 0.0116	0.0355 ± 0.0148 $	0.0399 ± 0.0147 †
**Uqcrb**	0.0507 ± 0.0118	0.0457 ± 0.0082	0.0353 ± 0.0072 $	0.0524 ± 0.0104	0.0624 ± 0.0120	0.0634 ± 0.0108
**Uqcrh**	0.0198 ± 0.0048	0.0260 ± 0.0035 $	0.0272 ± 0.0019 $	0.0291 ± 0.0047	0.0283 ± 0.0030	0.0327 ± 0.0026
**Uqcrq**	0.0289 ± 0.0045	0.0553 ± 0.0072	0.0262 ± 0.0055	0.1061 ± 0.0176	0.1668 ± 0.0635 $	0.0609 ± 0.0158 ‡
**Uqcrfs1**	0.0226 ± 0.0066	0.0316 ± 0.0116	0.0277 ± 0.0050	0.0351 ± 0.0108	0.0324 ± 0.0104	0.0323 ± 0.0099
**Uqcr10**	0.0183 ± 0.0079	0.0155 ± 0.0047	0.0240 ± 0.0078	0.0322 ± 0.0140	0.0332 ± 0.0108	0.0399 ± 0.0177
**Uqcr11**	0.0221 ± 0.0062	0.0167 ± 0.0084	0.0394 ± 0.0058 ‡	0.0333 ± 0.0098	0.0443 ± 0.0155	0.0274 ± 0.0125 †
**Uqcc2**	0.0472 ± 0.0061	0.0580 ± 0.0037	0.0615 ± 0.0064	0.0508 ± 0.0054	0.0411 ± 0.0052	0.0515 ± 0.0042
**Ttc19**	0.0129 ± 0.0047	0.0082 ± 0.0022	0.0167 ± 0.0041 †	0.0101 ± 0.0043	0.0103 ± 0.0041	0.0130 ± 0.0072
**Mtco1**	0.0563 ± 0.0049	0.0593 ± 0.0124	0.0602 ± 0.0037	0.0496 ± 0.0052	0.0633 ± 0.0058 $	0.0576 ± 0.0040
**Mtco2**	0.0283 ± 0.0053	0.0281 ± 0.0047	0.0269 ± 0.0041	0.0340 ± 0.0042	0.0403 ± 0.0188	0.0338 ± 0.0067
**Cox4i1**	0.0356 ± 0.0057	0.0305 ± 0.0054	0.0376 ± 0.0040	0.0394 ± 0.0084	0.0408 ± 0.0064	0.0415 ± 0.0060
**Cox5a**	0.0393 ± 0.0052	0.0403 ± 0.0080	0.0352 ± 0.0029	0.0566 ± 0.0044	0.0387 ± 0.0071 $	0.0553 ± 0.0043 †
**Cox5b**	0.0258 ± 0.0046	0.0235 ± 0.0085	0.0266 ± 0.0059	0.0290 ± 0.0077	0.0285 ± 0.0094	0.0321 ± 0.0072
**Cox6a2**	0.0135 ± 0.0015	0.0176 ± 0.0050 $	0.0226 ± 0.0025 ‡	0.0189 ± 0.0031	0.0165 ± 0.0019	0.0244 ± 0.0016 ‡
**Cox6b1**	0.0369 ± 0.0105	0.0436 ± 0.0139	0.0369 ± 0.0075	0.0448 ± 0.0191	0.0384 ± 0.0191	0.0483 ± 0.0133
**Cox6c**	0.0316 ± 0.0024	0.0259 ± 0.0024 $	0.0336 ± 0.0015 †	0.0386 ± 0.0024	0.0366 ± 0.0035	0.0354 ± 0.0033
**Atp5a1**	0.0296 ± 0.0051	0.0276 ± 0.0049	0.0311 ± 0.0055	0.0357 ± 0.0051	0.0390 ± 0.0094	0.0413 ± 0.0061
**Atp5b**	0.0319 ± 0.0046	0.0328 ± 0.0052	0.0346 ± 0.0077	0.0365 ± 0.0062	0.0326 ± 0.0149	0.0429 ± 0.0121
**Atp5c1**	0.0182 ± 0.0046	0.0166 ± 0.0083	0.0213 ± 0.0077	0.0249 ± 0.0084	0.0351 ± 0.0215	0.0262 ± 0.0098
**Atp5d**	0.0252 ± 0.0048	0.0227 ± 0.0058	0.0269 ± 0.0042	0.0337 ± 0.0049	0.0281 ± 0.0071	0.0325 ± 0.0040
**Atp5e**	0.0000 ± 0.0000	0.0088 ± 0.0044 $	0.0079 ± 0.0029 $	0.0080 ± 0.0040	0.0120 ± 0.0060	0.0047 ± 0.0023 †
**Atp5f1**	0.0184 ± 0.0080	0.0121 ± 0.0052	0.0185 ± 0.0065	0.0140 ± 0.0057	0.0144 ± 0.0073	0.0214 ± 0.0085
**Atp5h**	0.0252 ± 0.0097	0.0262 ± 0.0113	0.0297 ± 0.0068	0.0263 ± 0.0135	0.0427 ± 0.0237	0.0365 ± 0.0167
**Atp5i**	0.0134 ± 0.0047	0.0085 ± 0.0040	0.0120 ± 0.0053	0.0208 ± 0.0085	0.0189 ± 0.0123	0.0153 ± 0.0070
**Atp5j2**	0.0204 ± 0.0045	0.0197 ± 0.0089	0.0172 ± 0.0037	0.0218 ± 0.0076	0.0221 ± 0.0088	0.0237 ± 0.0092
**Atp5l**	0.0396 ± 0.0079	0.0408 ± 0.0092	0.0381 ± 0.0069 $	0.0409 ± 0.0141	0.0382 ± 0.0099	0.0513 ± 0.0116 ‡
**Atp5o**	0.0004 ± 0.0002	0.0063 ± 0.0027 $	0.0123 ± 0.0036 ‡	0.0064 ± 0.0016	0.0099 ± 0.0044	0.0207 ± 0.0033 ‡

Supplemental Table 2:The relative abundance of mitochondrial ETS proteins in the gastroc. Data were analyzed by two-way ANOVA (group x treatment) and are represented as mean from 5-7 rats per group. Data are in mean ± SD where $ indicates a significant difference from CON, † indicates a significant difference from MET and ‡ indicates significant differences from CON and MET.
