# Supplementary material for: Metformin treatment results in distinctive skeletal muscle mitochondrial remodeling in rats with different intrinsic aerobic capacities
Source: Aging Cell. 2024 Jun 24;23(9):e14235. doi: 10.1111/acel.14235 (PMC11488331; doi:10.1111/acel.14235)
Supplement: Supplementary file 8 — Table S3. [file ACEL-23-e14235-s006.pdf]

Soleus Synthesis Rates (k(1/day))												
	HCR Con		HCR MET		HCR WO		LCR Con		LCR MET		LCR WO	
Ndufa2	0.0005 ± 0.000		0.0000 ± 0.000		0.0259 ± 0.007	‡	0.0000 ± 0.000		0.0124 ± 0.006	$	0.0304 ± 0.015	‡
Ndufa4	0.0867 ± 0.014		0.0897 ± 0.016		0.0866 ± 0.013		0.1021 ± 0.033		0.1131 ± 0.025		0.1003 ± 0.020	
Ndufa5	0.0448 ± 0.022		0.0321 ± 0.010		0.0257 ± 0.004		0.0462 ± 0.004		0.0434 ± 0.026		0.0306 ± 0.007	
Ndufa6	0.1138 ± 0.006		0.1115 ± 0.008		0.1225 ± 0.009		0.0685 ± 0.008		0.1285 ± 0.015	$	0.1221 ± 0.011	$
Ndufa7	0.0575 ± 0.005		0.0724 ± 0.008	$	0.1020 ± 0.016	‡	0.0773 ± 0.014		0.0947 ± 0.011	$	0.1140 ± 0.006	‡
Ndufa8	0.0273 ± 0.008		0.0199 ± 0.008		0.0274 ± 0.010		0.0207 ± 0.009		0.0254 ± 0.011	$	0.0299 ± 0.008	†
Ndufa9	0.0747 ± 0.020		0.0678 ± 0.025		0.0628 ± 0.017		0.0612 ± 0.020		0.1220 ± 0.068		0.0743 ± 0.021	
Ndufa10	0.0586 ± 0.010		0.0655 ± 0.015		0.0670 ± 0.011		0.0654 ± 0.034		0.0643 ± 0.015		0.0664 ± 0.014	
Ndufa11	0.0795 ± 0.012		0.1179 ± 0.024	$	0.1033 ± 0.011		0.1218 ± 0.013		0.0857 ± 0.032	$	0.1179 ± 0.009	†
Ndufa12	0.0421 ± 0.008		0.0591 ± 0.008	$	0.0499 ± 0.004		0.0431 ± 0.006		0.0673 ± 0.011	$	0.0624 ± 0.009	$
Ndufa13	0.0627 ± 0.007		0.0751 ± 0.019		0.0489 ± 0.008	†	0.0594 ± 0.008		0.0686 ± 0.017		0.0766 ± 0.006	$
Ndufb5	0.0299 ± 0.004		0.0294 ± 0.004		0.0381 ± 0.004	‡	0.0296 ± 0.004		0.0543 ± 0.005	$	0.0460 ± 0.004	‡
Ndufb7	0.0509 ± 0.008		0.0516 ± 0.012		0.0789 ± 0.015	‡	0.0691 ± 0.013		0.0755 ± 0.017		0.0912 ± 0.024	$
Ndufb10	0.0302 ± 0.015		0.0156 ± 0.015		0.0270 ± 0.018		0.0256 ± 0.025		0.0377 ± 0.020		0.0309 ± 0.011	
Ndufb11	0.0285 ± 0.004		0.0258 ± 0.004		0.0334 ± 0.002	†	0.0226 ± 0.004		0.0321 ± 0.004	$	0.0357 ± 0.002	$
Ndufc2	0.0409 ± 0.004		0.0330 ± 0.004	$	0.0414 ± 0.004	‡	0.0327 ± 0.004		0.0406 ± 0.007	$	0.0462 ± 0.003	‡
Nd1	0.0158 ± 0.003		0.0251 ± 0.010		0.0325 ± 0.010	$	0.0157 ± 0.002		0.0297 ± 0.017	$	0.0352 ± 0.012	$
Nd4	0.0074 ± 0.004		0.0035 ± 0.002		0.0173 ± 0.009	‡	0.0000 ± 0.000		0.0000 ± 0.000		0.0134 ± 0.007	‡
Nd5	0.0284 ± 0.007		0.0312 ± 0.010		0.0486 ± 0.002	‡	0.0345 ± 0.005		0.0324 ± 0.009		0.0519 ± 0.001	‡
Ndufs1	0.0633 ± 0.008		0.0590 ± 0.011		0.0663 ± 0.009		0.0552 ± 0.007		0.0694 ± 0.010	$	0.0759 ± 0.011	$
Ndufs2	0.0318 ± 0.003		0.0398 ± 0.007	$	0.0433 ± 0.007	$	0.0374 ± 0.004		0.0359 ± 0.005		0.0498 ± 0.004	‡
Ndufs3	0.0542 ± 0.006		0.0580 ± 0.008		0.0602 ± 0.009		0.0528 ± 0.006		0.0702 ± 0.012	$	0.0652 ± 0.012	$
Ndufs5	0.0485 ± 0.011		0.0366 ± 0.009		0.0579 ± 0.005	†	0.0505 ± 0.007		0.0562 ± 0.012		0.0576 ± 0.010	
Ndufs6	0.0803 ± 0.008		0.1198 ± 0.031	$	0.0802 ± 0.015	†	0.0877 ± 0.010		0.0824 ± 0.023		0.0933 ± 0.010	
Ndufs7	0.0838 ± 0.022		0.0919 ± 0.012		0.1090 ± 0.023		0.0799 ± 0.020		0.1026 ± 0.035		0.1027 ± 0.018	
Ndufs8	0.0468 ± 0.005		0.0409 ± 0.007		0.0466 ± 0.003		0.0504 ± 0.007		0.0542 ± 0.006		0.0481 ± 0.004	
Ndufv1	0.0654 ± 0.005		0.0463 ± 0.012		0.0743 ± 0.006		0.0797 ± 0.025		0.0932 ± 0.006	$	0.0720 ± 0.010	
Ndufv2	0.0735 ± 0.013		0.0780 ± 0.015		0.0693 ± 0.012		0.0658 ± 0.010		0.0933 ± 0.017	$	0.0752 ± 0.007	†
SdhA	0.0602 ± 0.014		0.0608 ± 0.011		0.0688 ± 0.010		0.0621 ± 0.012		0.0682 ± 0.017		0.0741 ± 0.011	
SdhB	0.0822 ± 0.029		0.0714 ± 0.017		0.0731 ± 0.010		0.0494 ± 0.012		0.0686 ± 0.023		0.0773 ± 0.013	
SdhC	0.0512 ± 0.013		0.0518 ± 0.023		0.0604 ± 0.011		0.0536 ± 0.019		0.0509 ± 0.016		0.0525 ± 0.008	
Cyc1	0.0485 ± 0.002		0.0466 ± 0.003		0.0529 ± 0.005		0.0421 ± 0.008		0.0512 ± 0.008		0.0499 ± 0.007	
Uqcrc1	0.0220 ± 0.008		0.0147 ± 0.011		0.0261 ± 0.008		0.0257 ± 0.009		0.0312 ± 0.013		0.0285 ± 0.011	
Uqcrc2	0.0295 ± 0.008		0.0245 ± 0.013		0.0339 ± 0.007		0.0431 ± 0.015		0.0492 ± 0.018		0.0378 ± 0.006	
Uqcrb	0.0267 ± 0.002		0.0268 ± 0.002		0.0377 ± 0.002	‡	0.0283 ± 0.003		0.0357 ± 0.004	$	0.0357 ± 0.003	$
Uqcrh	0.0432 ± 0.002		0.0391 ± 0.005		0.0507 ± 0.003	‡	0.0476 ± 0.007		0.0583 ± 0.006	$	0.0465 ± 0.004	‡
Uqcrfs1	0.0334 ± 0.007		0.0527 ± 0.009	$	0.0406 ± 0.008		0.0459 ± 0.012		0.0568 ± 0.011		0.0537 ± 0.014	
Uqcr10	0.0588 ± 0.022		0.0269 ± 0.019		0.0486 ± 0.016		0.0462 ± 0.021		0.0767 ± 0.060		0.0769 ± 0.027	
Uqcr11	0.0467 ± 0.010		0.0292 ± 0.014		0.0572 ± 0.010		0.0799 ± 0.008		0.0000 ± 0.000		0.0503 ± 0.013	
Mtco1	0.0604 ± 0.029		0.0605 ± 0.051		0.0606 ± 0.017		0.0323 ± 0.024		0.0674 ± 0.036		0.0673 ± 0.023	
Mtco2	0.0306 ± 0.008		0.0359 ± 0.008		0.0355 ± 0.006		0.0341 ± 0.010		0.0479 ± 0.013		0.0406 ± 0.009	
Cox4i1	0.0572 ± 0.007		0.0629 ± 0.012		0.0706 ± 0.010		0.0568 ± 0.009		0.0668 ± 0.012		0.0679 ± 0.012	
Cox5a	0.0118 ± 0.006		0.0019 ± 0.001	$	0.0096 ± 0.005	†	0.0155 ± 0.006		0.0035 ± 0.002	$	0.0000 ± 0.000	‡
Cox5b	0.0292 ± 0.014		0.0318 ± 0.011		0.0323 ± 0.014		0.0275 ± 0.011		0.0436 ± 0.013		0.0340 ± 0.011	
Cox6b1	0.0531 ± 0.022		0.0550 ± 0.018		0.0543 ± 0.020		0.0492 ± 0.022		0.0677 ± 0.025		0.0706 ± 0.031	
Cox6c	0.0680 ± 0.016		0.0568 ± 0.019		0.0547 ± 0.009		0.0505 ± 0.005		0.0462 ± 0.006		0.0657 ± 0.005	†
Atp5a1	0.0494 ± 0.009		0.0478 ± 0.018		0.0539 ± 0.010		0.0402 ± 0.009		0.0610 ± 0.025		0.0618 ± 0.011	‡
Atp5b	0.0270 ± 0.003		0.0369 ± 0.008		0.0337 ± 0.008		0.0276 ± 0.009		0.0537 ± 0.015	$	0.0388 ± 0.009	
Atp5c1	0.0462 ± 0.012		0.0421 ± 0.022		0.0408 ± 0.014		0.0433 ± 0.017		0.0392 ± 0.011		0.0418 ± 0.012	
Atp5d	0.0287 ± 0.006		0.0258 ± 0.012		0.0309 ± 0.006		0.0266 ± 0.008		0.0479 ± 0.017	$	0.0416 ± 0.006	$
Atp5f1	0.0318 ± 0.002		0.0318 ± 0.002		0.0325 ± 0.002		0.0255 ± 0.002		0.0378 ± 0.002	$	0.0362 ± 0.003	$
Atp5h	0.0329 ± 0.008		0.0371 ± 0.017		0.0330 ± 0.008		0.0384 ± 0.009		0.0502 ± 0.020		0.0448 ± 0.006	
Atp5i	0.0245 ± 0.015		0.0279 ± 0.025		0.0195 ± 0.017		0.0258 ± 0.013		0.0348 ± 0.029		0.0244 ± 0.010	
Atp5j2	0.0365 ± 0.005		0.0289 ± 0.005	$	0.0348 ± 0.004	†	0.0310 ± 0.005		0.0449 ± 0.003	$	0.0386 ± 0.003	‡
Atp5l	0.0627 ± 0.015		0.0469 ± 0.010	$	0.0580 ± 0.009		0.0415 ± 0.005		0.0631 ± 0.014	$	0.0669 ± 0.010	$
Atp5o	0.0326 ± 0.005		0.0406 ± 0.007		0.0409 ± 0.008		0.0352 ± 0.006		0.0506 ± 0.015	$	0.0467 ± 0.006	$

Supplemental Table 3:The relative abundance of mitochondrial ETS proteins in the soleus. Data were analyzed by two-way ANOVA (group x treatment) and are represented as mean from 5-7 rats per group. Data are in mean ± SD where $ indicates a significant difference from CON, † indicates a significant difference from MET and ‡ indicates significant differences from CON and MET.
